# Nanohybrid layered double hydroxide materials as efficient catalysts for methanol electrooxidation†

**DOI:** 10.1039/c9ra01270b

**Published:** 2019-05-01

**Authors:** Shimaa Gamil, Waleed M. A. El Rouby, Manuel Antuch, I. T. Zedan

**Affiliations:** Renewable Energy Science and Engineering Department, Faculty of Postgraduate Studies for Advanced Science, Beni-Suef University 62511 Beni-Suef Egypt; Materials Science and Nanotechnology Department, Faculty of Postgraduate Studies for Advanced Science, Beni-Suef University 62511 Beni-Suef Egypt waleedmohamedali@psas.bsu.edu.eg; Paris-Sud University, ICMMO-Eriée, UMR CNRS 8182 91405 Orsay France

## Abstract

In this work, efficient methanol oxidation fuel cell catalysts with excellent stability in alkaline media have been synthesized by including transition metals to the layered double hydroxide (LDH) nanohybrids. The nanohybrids CoCr-LDH, NiCoCr-LDH and NiCr-LDH were prepared by co-precipitation and their physicochemical characteristics were investigated using TEM, XRD, IR and BET analyses. The nanohybrid CoCr-LDH is found to have the highest surface area of 179.87 m^2^ g^−1^. The electrocatalytic activity measurements showed that the current density was increased by increasing the methanol concentration (from 0.1 to 3 M) as a result of its increased oxidation at the surface. The nanohybrid NiCr-LDH, showing the highest pore size (55.5 Å) showed the highest performance for methanol oxidation, with a current density of 7.02 mA cm^−2^ at 60 mV s^−1^ using 3 M methanol. In addition, the corresponding onset potential was 0.35 V (at 60 mV s^−1^ using 3 M methanol) which is the lowest value among all other used LDH nanohybrids. Overall, we observed the following reactivity order: NiCr-LDH > NiCoCr-LDH > CoCr-LDH, as derived from the impedance spectroscopy analysis.

## Abstract 2

Methanol electrooxidation over layered double hydroxides.

## Graphical abstract

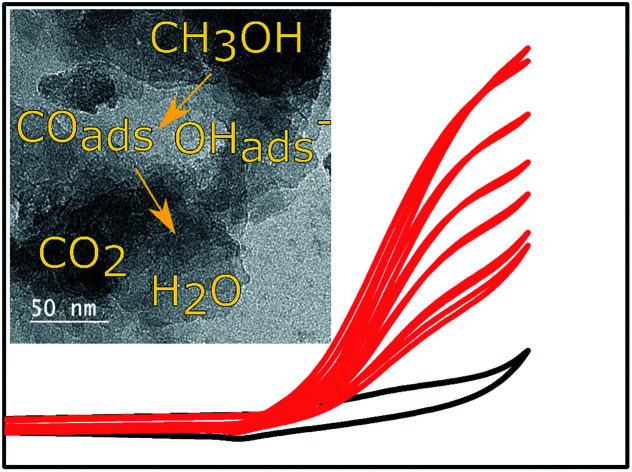

## 1. Introduction

Scientific research to find convenient alternatives to fossil energy is an important research topic worldwide. The research interest in green energy technologies is reflected by the use of air and water for electric power generation through fuel cell technology. Fuel cells have received much attention for power generation compared to other conventional energy storage systems because of their unique characteristics such as low cost, diminished greenhouse gas emission, smog pollution and simple structure.^1–3^

Direct methanol fuel cells (DMFCs) offer wide possibilities to power electric vehicles and electronic portable devices.^4,5^ The use of methanol in fuel cells is attractive due to its high energy density, ease of storage, preparation, and availability.^6,7^ DMFCs are mainly dependent on the electrocatalytic activity of the electrode materials.^6,8,9^ The main problems which reduce its power output and efficiency are the high cost and the high tendency to poisoning by intermediate adsorption at the surface which blocks the active sites.^10,11^ Platinum based catalysts have a unique electrocatalytic activity for promoting methanol oxidation.^12,13^ However, they are very expensive which limits their use.^14^ Therefore, current research has been directed to improve the activity of methanol electro-oxidation catalysts with reduced precious metals.^9,15^

Nanomaterials are interesting in the field of electrochemistry due to their high surface area to volume ratio, unique structure and promising physicochemical characteristics.^10,16,17^ Layered double hydroxides (LDHs) are formed by layers of metallic hydroxides which are separated by hydrated anions as interlayer.^16,18,19^ These LDHs have been extensively studied for different applications as new layered inorganic nanomaterials which are two-dimensional organized structures.^10,16,18,20–22^ Moreover, these are ionic solids with excellent chemical and thermal stability which obviously drive them as alternative materials for catalyzing methanol oxidation.^11^ The modification of electrodes by using LDHs is a new strategy for catalyzing methanol oxidation. It has been shown that LDH compounds bearing combinations of divalent and trivalent transition metals in their structure were promising in electrochemistry.^17,23^ The improvement of the intrinsic properties of LDHs rely on the identity of cations composing the layers.^24^ Among the prospective transition metals, cobalt is an attractive choice for using in electrochemical applications due to its low cost, good thermal conductivity and environmental friendliness.^25,26^ Co-based materials have introduced non-precious and effective catalysts for the oxygen evolution reaction. Particularly, the Cr^3+^ cation has a special electronic configuration, which enhances charge transfer and electron capture.^27^ Chenlong Dong *et al.*, used the binary CoCr LDH as a novel and an excellent electroactive catalyst with long-term stability.^28^ The use of nickel within the LDHs structure gained substantial interest from the scientific view due to its electrochemical reactivity, especially towards the electrocatalytic oxidation of alcohols.^25,29,30^ Typically, nickel has a one-electron reversible redox couple, Ni(iii)/Ni(ii), which is desirable for its electrocatalytic activity.^1^ Y. Vlamidis *et al.* demonstrated that Ni/Fe LDHs can achieve higher performance for methanol oxidation as compared to Ni/Al LDHs. They proposed that Fe can enhance the electroactivity of Ni by increasing the number of active sites in the Fe containing material.^31^ It was found that the incorporation of Cr^3+^ into Co or Ni hydroxides enhances the conductivity.^27^ Furthermore, Cr^3+^ ions can be oxidized easily to higher oxidation states during the methanol oxidation reaction, whose effect is positive on the obtained current density value. Recently, Y. Wen *et al.* used NiCr-LDH, for the first time, as an efficient and stable bifunctional catalyst for the electrocatalytic oxidation and reduction of water.^27^ Thus, the continuous challenges to develop promising transition-metal LDH electrode materials for methanol oxidation by increasing their performance and stability is essential nowadays. In this work we have prepared three mesostructured nanohybrids CoCr-LDH, NiCoCr-LDH and NiCr-LDH by a coprecipitation method, which have not been studied towards direct oxidation methanol before. As a result, the gradual replacement of cobalt by nickel may promote higher electrocatalytic activity and better stability for methanol oxidation. This due to the electrochemical reactivity of nickel towards methanol oxidation. Notably, compared with other prepared nanohybrids, the nanohybrid NiCr-LDH showed the fast electrode kinetics (lowest value of the charge transfer resistance *R*_CT_) and the highest current density value. Therefore, this work not only presents new types of LDHs as promising cheap catalysts, but also provides new insights into the role of nickel ions for enhancing the electrochemical activity of the LDH layer.

## 2. Materials and methods

### 2.1. Materials

Cobalt(ii) nitrate hexahydrate (Co(NO_3_)_2_·6H_2_O), nickel(ii) nitrate hexahydrate (Ni(NO_3_)_2_·6H_2_O), chromium(iii) nitrate nonahydrate (Cr(NO_3_)_3_·9H_2_O), sodium carbonate (Na_2_CO_3_), methanol (MeOH) and sodium hydroxide (NaOH) were purchased from Oxford company Mumbai-400002 (India). The reagents described herein were of analytical grade; these were employed without additional treatment. Deionized (DI) water was used for all syntheses and treatment processes under ambient conditions.

### 2.2. Synthesis of LDHs nanohybrids

The three nanohybrids LDHs were prepared by a co-precipitation method.^32,33^ For binary CoCr-LDH and NiCr-LDH nanohybrids the mole ratio of the nitrate salts was 2 : 1, while for the ternary NiCoCr-LDH nanohybrid the mole ratio was 1 : 1 : 1. Briefly, 200 mL of a solution containing 1 M NaOH and 0.01 M Na_2_CO_3_ was first prepared; then, 100 mL of the nitrate salts were added dropwise under continuous mechanical stirring. After these additions, the obtained suspensions were aged for 16 h at 65 °C; afterwards these were cooled to ambient temperature. The obtained products were separated by filtration and washed several times using DI water. Finally, all LDHs were dried out at 60 °C during additional 24 h. The prepared materials were termed as CoCr-LDH, NiCr-LDH and NiCoCr-LDH.

### 2.3. Characterization of LDH nanohybrids

The phase structure and crystallinity of the prepared catalysts were assessed by X-ray diffraction (XRD) in an X-ray diffractometer (PANalytical, Empyrean, Netherlands) equipped with CuK_α_ radiation of wavelength *λ* = 1.54045 Å and accelerating voltage of 40 kV with operating current of 35 mA. Transmission electron microscopy (TEM) (JEOL JEM-2100) was used to study the morphologies of the prepared LDH samples. Fourier transform infrared (FTIR) spectroscopy was performed to gain information on the intercalation nature of the nanohybrids LDH using Vertex 70 (Bruker, Germany). The surface areas and pore-size distributions of the nanohybrids under investigations were calculated from N_2_ adsorption–desorption isotherms determined with a Tri-Star II 3020 (Micromeritics, USA) analyzer by Brunauer–Emmett–Teller (BET) method.

### 2.4. Electrode preparation for methanol oxidation

The glassy carbon (GC) electrodes with area of 0.0706 cm^2^ were cleaned by polishing with alumina fine powder on a smooth emery paper until it became like a mirror and then washed with distilled water followed by acetone. Then, the working electrode was prepared by sonicating the solution of 2 mg of the prepared catalyst (LDH) in 420 μL isopropanol and 20 μL Nafion (5%) solution for 30 min at room temperature. Subsequently, 15 μL of the prepared slurry was spread over the active area of the GC electrode. The GC surface was then dried out till ambient temperature prior electrochemical tests.

### 2.5. Electrocatalytic activity measurements

The electrocatalytic activities of the prepared catalysts toward methanol oxidation in alkaline media were carried out in a three-electrode cell configuration at room temperature, using a Ag|AgCl|Cl^−^ (KCl solution of 3.0 M) reference electrode, a Pt wire as counter electrode, and working electrode (the GC electrode prepared by the above-mentioned procedure). The electrolyte solution was 1 M KOH. All electrochemical experiments were performed using Metrohm-Autolab potentiostat controlled by a computer *via* NOVA 1.11 software. Cyclic voltammetry (CV) at scan rates from 10 to 100 mV s^−1^, electrochemical impedance spectroscopy (EIS) at 0.6 V and amplitude of 10 mV, linear sweep voltammetry (LSV) at 60 mV s^−1^ and chronoamperometry (CA) at 0.6 V measurements were studied.

## 3. Results and discussion

### 3.1. Characterization of the prepared LDH nanohybrids

**Fig. 1:**
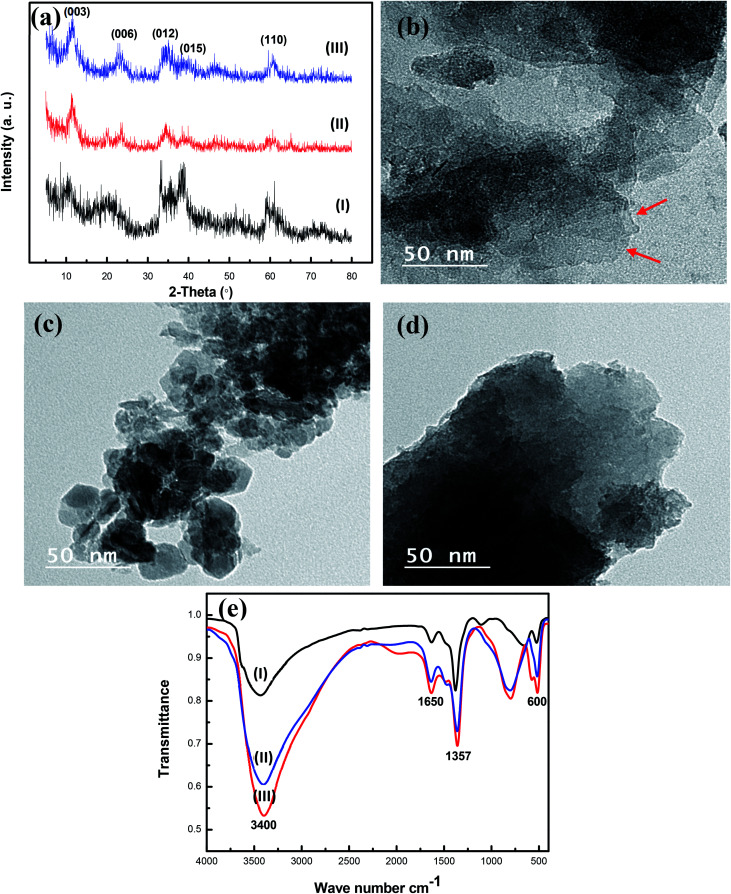
(a) The XRD patterns for nanohybrid (I) NiCr-LDH, (II) CoCr-LDH and (III) NiCoCr-LDH; TEM images of hybrid (b) NiCr-LDH, (c) CoCr-LDH and (d) NiCoCr-LDH; (e) FTIR spectra of hybrid (I) NiCr-LDH, (II) NiCoCr-LDH and (III) CoCr-LDH.

The X-ray diffraction analysis of the prepared nanohybrid CoCr-LDH, NiCr-LDH, and NiCoCr-LDH was carried out to confirm their crystallographic structures along with the purity of the obtained phases. The XRD patterns as shown in Fig. 1a indicates that the main characteristic peaks of the prepared nanohybrid LDHs matches with the expected profile for LDH compounds with crystallographic planes (003), (006), (012), (015), (110) and (113) observed at 2*θ* values of 11.1°, 22.2°, 34.0°, 38.8° and 59.4° respectively, indicating that the synthesized LDHs were with well lamellar structures. The slight differences in broadness in the Bragg diffraction peaks among the different LDH are likely to be due to differences in their crystallinity. The remaining X-ray diffraction peaks in CoCr-LDH, NiCoCr-LDH and NiCr-LDH related to Co, Ni and Cr doped oxides.

The structural features of the synthesized nanohybrids CoCr-LDH, NiCoCr-LDH and NiCr-LDH were observed using TEM images as shown in Fig. 1b–d. It demonstrates that the prepared nanohybrids have flake-like morphology with pure sets of small particles of hexagonal ordered. This structure with cross linked nanoflakes yield the high porosity exhibited by the prepared nanohybrids. Moreover, it is clear from Fig. S1† that, the NiCr-LDH nanohybrid with a much lower aggregation than CoCr-LDH and NiCoCr-LDH which should contribute to the NiCr-LDH electrocatalytic activity.

FTIR spectroscopy was performed to assess the presence of each functional group and both the interlayer anions and cations in the LDHs as shown in Fig. 1e. The bands appearing at low wavenumbers (*i.e.*, below 800 cm^−1^) were essentially due to both the stretching and bending normal modes of M–O–H and O–M–O (M = metal) in brucite-like layers.^28,34^ The band which appeared at 1357 cm^−1^ confirmed the presence of CO_3_^−2^ group.^33^ Obviously strong absorption bands at 1650 and 3400 cm^−1^ for the prepared nanohybrid LDHs can be attributed to the stretching mode of the OH group of water molecules inside the interlayer of LDH and the broadness of such band indicates a variety of hydrogen bonds at the surface of the prepared nanohybrids. The intensity of the band at 1650 and 3400 cm^−1^ varied from high to low according to the hybrid, the intensity of CoCr-LDH more than that of NiCoCr-LDH and NiCr-LDH. This observation indicates that CoCr-LDH and NiCoCr-LDH have high surface adsorbed –OH group but NiCr LDH has higher amount of defects (oxygen vacancies).^35^

**Fig. 2:**
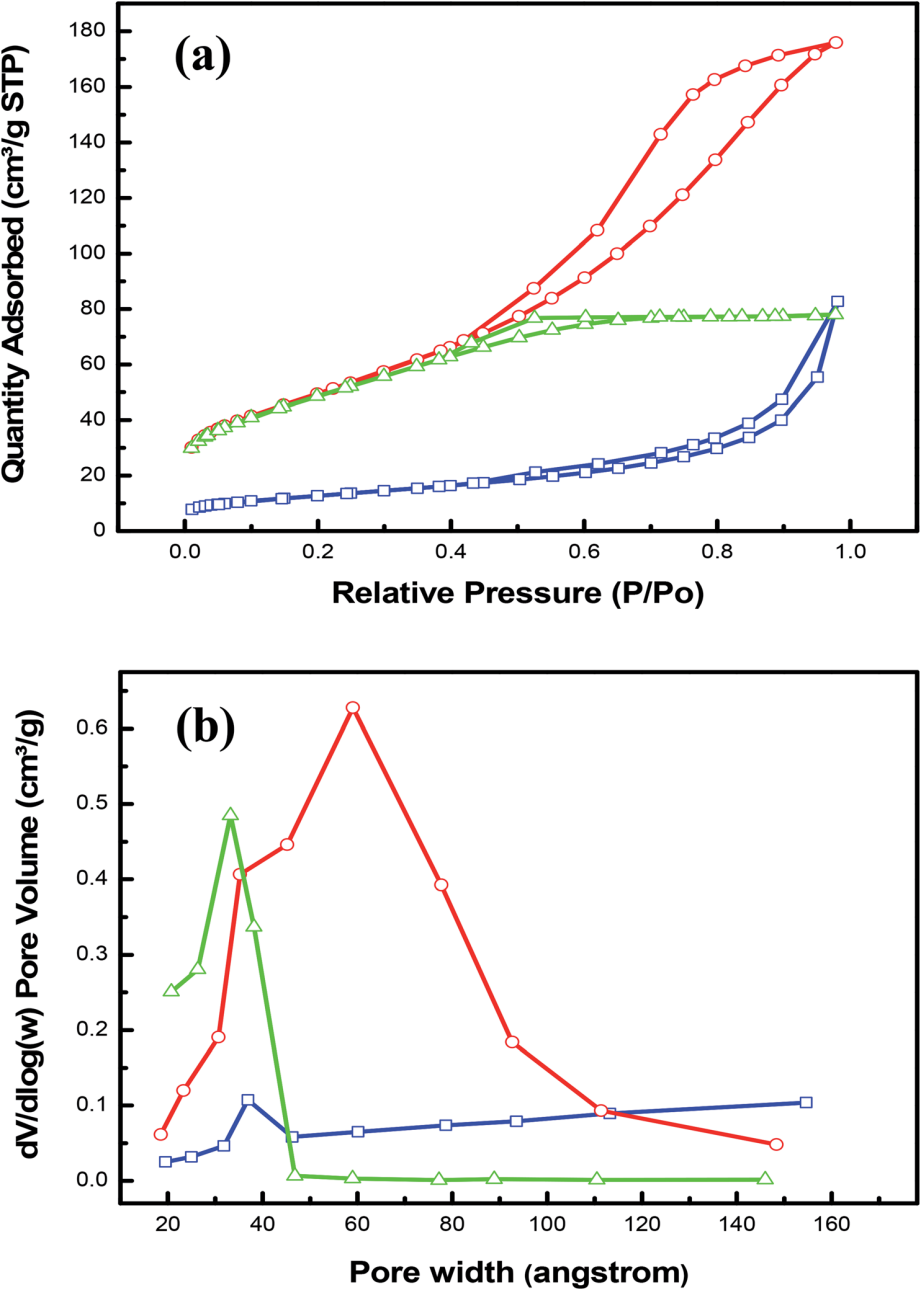
(a) N_2_ adsorption–desorption loops and (b) the BJH pore size distribution profiles of nanohybrid CoCr-LDH (red), NiCr-LDH (blue) and NiCoCr-LDH (green).

To further characterize the as-prepared nanohybrids LDHs, the nitrogen adsorption–desorption isotherms were obtained in order to study the surface area, the average pore size and the mesoporosity of the samples; which should have an influence on the electrochemical performance. Each sample showed a classical IV isotherm with a hysteresis loop (*P*/*P*_0_ > 0.4), suggesting the presence of mesopores as depicted in Fig. 2a. It is clear also from this figure that the CoCr-LDH has the highest specific surface area of 179.87 m^2^ g^−1^. This value is significantly larger than that of NiCoCr-LDH (174.62 m^2^ g^−1^) and that of NiCr-LDH (45.31 m^2^ g^−1^). From the obtained results, it was noticed that a H1 hysteresis loop is observed for NiCr-LDH hybrid, indicating the coexistence of high pore size and shape uniformity. The uniformity can provide efficient transport pathways for electrolyte ions and create high active sites for methanol oxidation.^36–40^ Moreover, as shown in the Table 1, the nanohybrid NiCr-LDH has the highest value of average pore width of 56 Å. The redox reactions efficiency of both CoCr-LDH and NiCoCr-LDH nanohybrids may be reduced since CoCr-LDH (51 Å) has large pore size diameter with non-uniform distribution while NiCoCr-LDH (27 Å) has narrow pore size distribution with uniform size. This explanation agrees with Z. Luojiang *et al.* who have found that the increase in pore volume is related to the formation of secondary pores which can enhance the diffusion process.^41^ In addition, it was clear from Fig. 2b that the pore size distribution of CoCr-LDH and NiCoCr-LDH was centered at 65 and 35 Å, respectively which could not serve for the efficient access of OH^–^ to participate in the redox reaction but the pore size distribution mainly centered at 38 Å for NiCr-LDH is optimal for the better OH^–^ diffusion towards reactive sites.^8,41^

**Table 1:** Specific BET surface area, pore volume and average pore size of the CoCr-LDH, NiCoCr-LDH and NiCr-LDH samples

Sample	NiCr LDH	CoCr LDH	NiCoCr LDH
BET surface area (m^2^ g^−1^)	45.31	179.87	174.62
A cumulative surface area of pores (m^2^ g^−1^)	42.03	192.79	160
A cumulative volume of pores (cm^3^ g^−1^)	0.06	0.24	0.11
Average pore width (Å)	55.5	50.51	27

### 3.2. Electrocatalytic activity

**Fig. 3:**
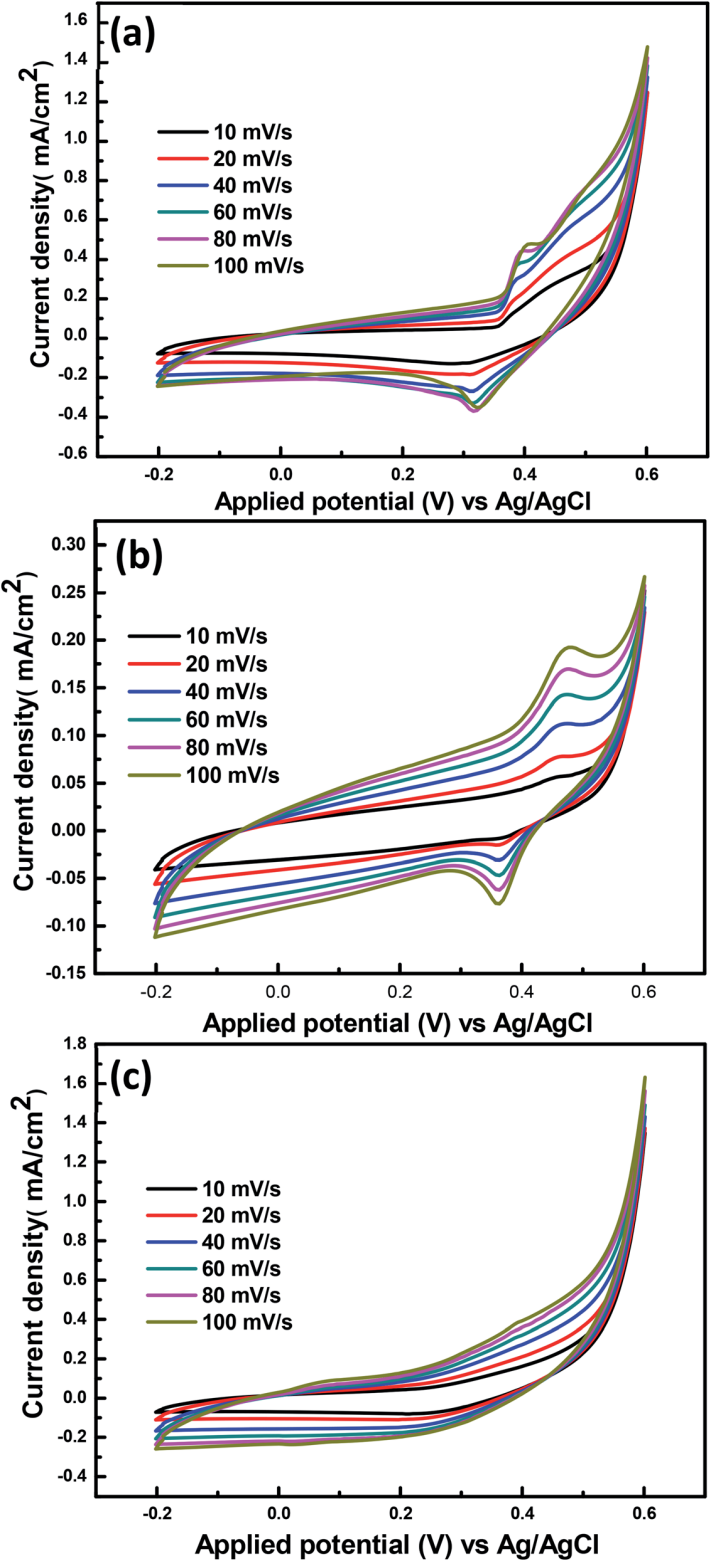
CVs of the (a) NiCr-LDH and (b) CoCr-LDH (c) NiCoCr-LDH in 1 M KOH at different scan rates at 25 °C.

A conventional three-electrode cell configuration was used to explore the catalytic performance of the prepared CoCr-LDH, NiCr-LDH and NiCoCr-LDH towards methanol oxidation. In order to evaluate the effect of the ohmic drop in our voltammetric measurements, we proceeded to determine the series resistance of the system by EIS, as the limit of *Z*_Re_ at high frequency (*R*_u_ = 1.5 Ω cm^2^), this value multiplied by the current densities obtained by CV (the highest value being around 6 mA cm^−2^) yields an estimated ohmic drop of 8 mV. Considering that this is a low value, further ohmic drop compensation was not performed. Current densities herein reported were determined per geometric surface area. The typical cyclic voltammetry of the prepared LDHs was carried out within the potential window from −200 to 600 mV (*vs.* Ag/AgCl) at room temperature and different scan rates (10, 20, 40, 60, 80 and 100 mV s^−1^). Fig. 3 shows the CV curves of these three nanohybrids LDHs in 1 M KOH electrolyte. Pairs of oxidation and reduction peaks are obtained in the anodic and cathodic sweeps in case of CoCr-LDH and NiCr-LDH nanohybrids. The increase of the current density was observed upon various potential sweeps, which implies the formation of an active layer atop the catalysts caused by CV cycling. This is likely to be related to the entry of hydroxyl anions inside the catalyst interlayer.^14,42^ On the other hand, NiCoCr-LDH hybrid showed a superior current density, which is observable from the results (Table 2). It is observed that at scan rate of 100 mV s^−1^, the current density values were 0.27, 1.48 and 1.63 mA cm^−2^ for CoCr-LDH, NiCr-LDH and NiCoCr-LDH, respectively. The NiCoCr-LDH hybrid exhibits a capacitive behavior and the enhanced electrochemical properties of it may be attributed to the transformation of the surface layer to a mixture of mixed metal oxyhydroxides as well as more active sites derived from the incorporation of the three transition metals.^43^ Together with mesoporous structure, pores distribution and high surface area (174.62 m^2^ g^−1^) which greatly improves the electrochemical performance.

**Table 2:** The current density of the CoCr-LDH, NiCoCr-LDH and NiCr-LDH samples at different scan rates with 1 M KOH

Scan rate (mV s^−1^)	Current density (mA cm^−2^)		
	CoCr LDH	NiCr LDH	NiCoCr LDH
10	0.25	1.25	1.35
20	0.23	1.25	1.37
40	0.24	1.33	1.43
60	0.25	1.38	1.49
80	0.26	1.42	1.56
100	0.27	1.48	1.63

**Fig. 4:**
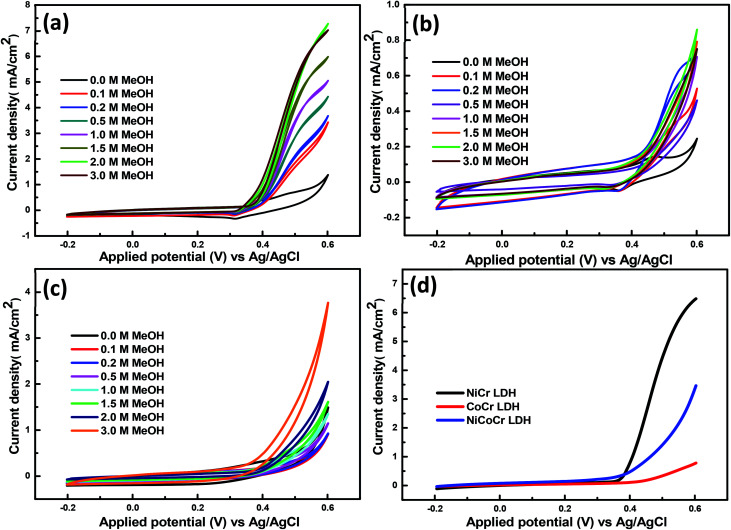
CVs of the nanohybrids at different concentration of CH_3_OH; (a) NiCr-LDH and (b) CoCr-LDH (c) NiCoCr-LDH; (d) the onset potentials for the nanohybrids with 3 M methanol concentration.

Fig. 4 displays the electrochemical performance of the obtained hybrid materials using diverse methanol concentrations (0.1, 0.2, 0.5, 1, 1.5, 2, 3 M). The electrochemical characteristics indicated that there is a progressive increase in the current density upon methanol addition, which indicates further methanol oxidation at the surface of the prepared hybrids. The methanol oxidation process may be followed during the cathodic half cycles given that both products or intermediates may be desorbed from the surface.^44^ Interestingly, there was a direct relationship between the electrocatalytic activity of the prepared nanohybrid LDHs for methanol oxidation and the nickel content. Whereas, the gradual substitution of cobalt by nickel in the LDH nanohybrid led to gradual increase in the current density. It was observed that the intense increase of the anodic current density was obtained during the measurements using 2 M methanol at scan rate of 60 mV s^−1^ for the catalyst NiCr-LDH. It was 3 times higher than NiCoCr-LDH and 8 times higher than CoCr-LDH (Fig. S2†). This is due to the low catalytic activity of cobalt as compared to nickel.^45^ The better performance of NiCr-LDH electrode (Fig. 4a) was attributed to the intrinsic activity of this compound, joined to its mesoporous structure and large pore width which enhance mass transport and electron transfer.^8^ Also, it has the high amount of the surface reactive oxygen species (according to FTIR results) directly participated in methanol oxidation. It is known from the literature that defective Ni(OH)_2_ species promotes the OH^–^ moving from Ni(OH)_2_ to the adjacent nanocrystals forming a new channels and active sites for OH^–^ adsorption and developing the removal of carbonaceous poisons (CO) from the catalyst surface which increase the electroactivity of nanohybrid LDH.^13^

The methanol oxidation performance was further studied by Linear Sweep Voltammetry (LSV). The onset potential is an important parameter to assess the effectiveness of the introduced electrocatalysts in alcohols oxidation because this is the point at which the reaction product is formed. In alcohol oxidation, a more negative onset potential for the anodic reaction means a lower over potential and hence a higher activity.^46^ There is an obvious change in the onset potential value before and after methanol addition (Fig. S3†). It shifted to more negative values by increasing the methanol concentration for the CoCr-LDH, NiCoCr LDH and NiCr-LDH which indicated the high electrocatalytic activity of them towards the methanol oxidation reaction. From Fig. 4d it can be inferred that the increase of Ni ratio in the LDH nanohybrid causes more negative onset potential, signifying an enhanced electrocatalytic methanol oxidation. Overall, the onset potential was 0.42, 0.38 and 0.35 V for CoCr-LDH, NiCoCr-LDH and NiCr-LDH, respectively using 3 M methanol at scan rate of 60 mV s^−1^.

**Fig. 5:**
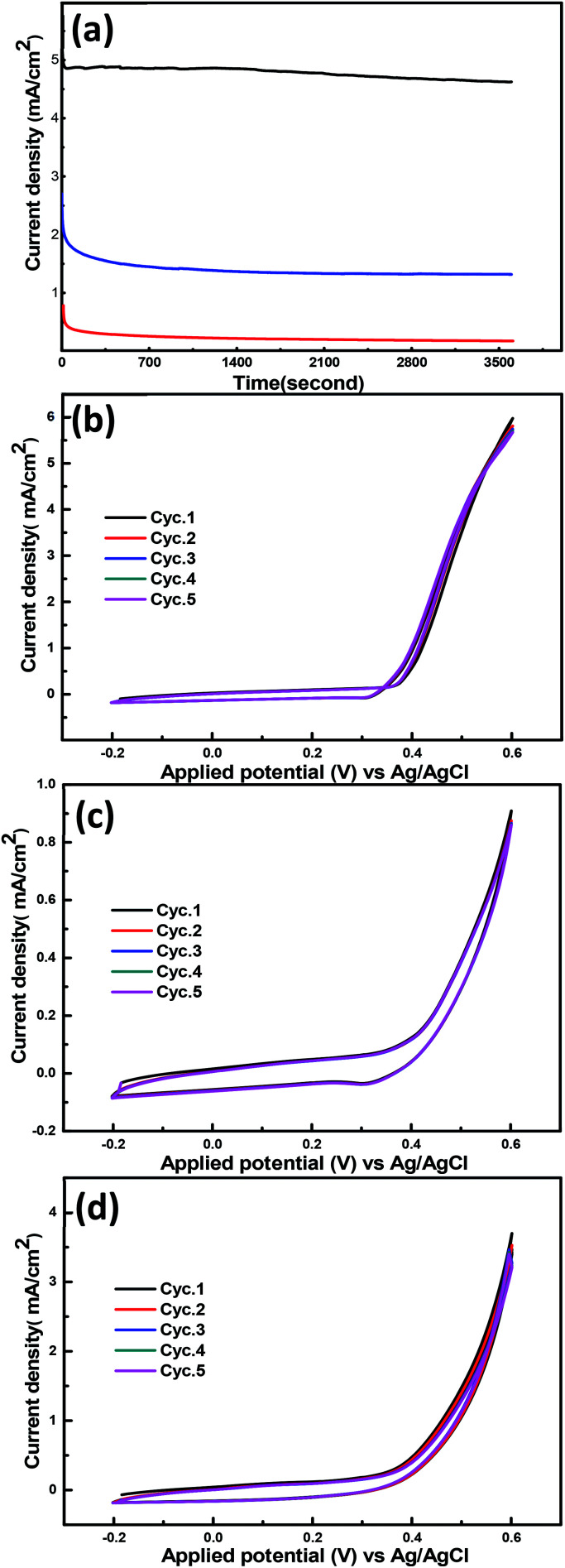
(a) Chronoamperometric response for the prepared hybrids NiCr-LDH (black), CoCr-LDH (red) and NiCoCr-LDH (blue). CVs for 5 cycles after stability of the prepared hybrids (b) NiCr-LDH (c) CoCr-LDH and (d) NiCoCr-LDH.

The electrochemical stability of the synthesized nanohybrid materials is another important issue to account for aiming at practical applications. Consequently, chronoamperometric measurements at a constant potential (600 mV *vs.* Ag/AgCl) in 1 M KOH and 3 M methanol were performed to assess the stability of these materials. It can be seen that the NiCr-LDH shows a superior durability as shown in Fig. 5a. Initially, the current density decreased rapidly due to the double layer contribution, and then decreased slowly with time, which is due to the formation of the carbonaceous intermediate CO_ads_.^47^ These results confirm the long-term stability of the NiCr-LDH, whose performance for methanol oxidation is superior if compared with the stability of the CoCr-LDH and the NiCoCr-LDH nanohybrids.

Fig. 5b–d shows 5 cyclic voltammograms recorded after the chronoamperometric stability tests. This behavior proves the outstanding stability of these materials since there is no appreciable change in the electrocatalytic activity.

### 3.3. Reaction dynamics studied by electrochemical impedance spectroscopy

Electrochemical Impedance Spectroscopy (EIS) consists in the study of the impedance variation of an electrochemical system with the frequency of a small-amplitude AC perturbation. It is used to characterize complex electrochemical reactions, along with faradaic and non-faradic processes.^48^

In alkaline media, the mechanistic details of methanol oxidation may be summarized by the following general equations^49,50^

1 M + OH^−^ → M–(OH)_ads_ + e^−^

2 M–(CH_3_OH)_ads_ + 4OH^−^ → M–(CO)_ads_ + 4H_2_O + 4e^−^

3 M–(CO)_ads_ + M–(OH)_ads_ + OH^−^ → 2M + CO_2_ + H_2_O + e^−^

where M represents an active metallic site at the surface of the electrode.

This mechanism consists in the oxidative adsorption of the hydroxyl ion (eqn (1)) followed by the oxidative adsorption and dehydrogenation of methanol to yield adsorbed carbon monoxide (eqn (2)). Finally, adsorbed intermediates may react in order to regenerate active catalytic sites and form carbon dioxide (eqn (3)).

**Fig. 6:**
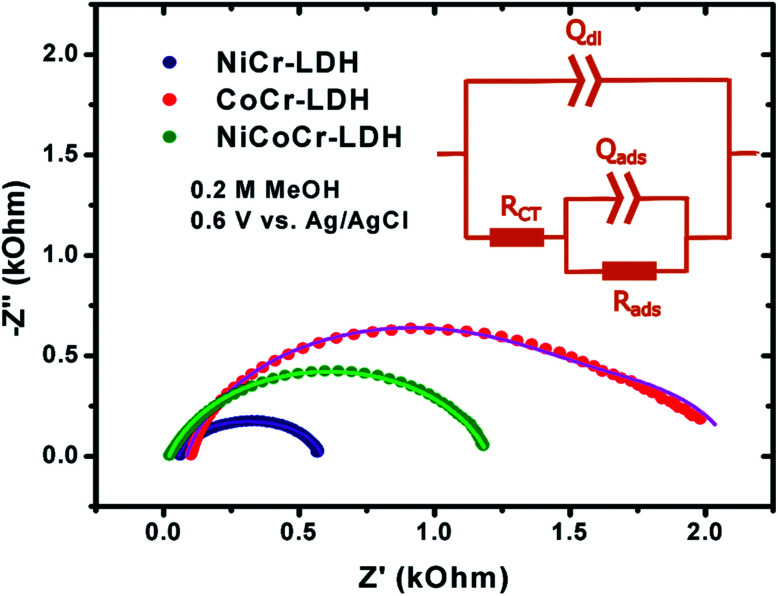
Nyquist plots for the oxidation reaction at 0.60 V using 2 M methanol (inset shows equivalent circuit compatible with the experimental impedance data for methanol oxidation on NiCr-LDH, CoCr-LDH and NiCoCr-LDH).

The EIS analysis of the CoCr-LDH, NiCoCr-LDH and NiCr-LDH nanohybrids were carried out at 600 mV *vs.* Ag/AgCl to examine the main behavior of these nanohybrids as electrodes using various methanol concentrations (0.1, 0.2, 0.5, 1, 1.5, 2 and 3 M, see Fig. S4–S6†). Typical impedance spectra are depicted in Fig. 6. It is worth noting that the mechanism of methanol oxidation is complex due to multiple electron transfer and adsorption/desorption steps which depend much on the nature of the electrocatalyst. Therefore, EIS spectra can display a large variety of features, including inductive loops, negative resistance, and multiple time constants.^44,49–61^ In our case, the spectra presented broad semicircles that could not be fit with a Randles circuit; instead, two relaxation constants could be identified, which has been observed before.^44^ Likewise, the semicircles were depressed, thus constant phase elements (CPEs) were included in the fit in order to account for surface heterogeneities detected with TEM microscopy. Such surface heterogeneities are responsible for uneven current lines at the interface which are described by the CPE. Consequently, the equivalent circuit depicted in Fig. 6 inset was used to model the experimental data. The circuit comprises two contributions: (i) one at high frequency, associated to the charge transfer rate (*R*_CT_) and the double layer capacitance (*Q*_dl_); and another contribution (ii) related to the rate of intermediate adsorption (*R*_ads_) and to the adsorption capacitance (*Q*_ads_).

Interestingly, none of the impedance spectra recorded in this work showed inductive characteristics. Certainly, inductive loops appear in the fourth quadrant of a Nyquist complex plot and have been thoroughly interpreted as a signature of the oxidation of (CO)_ads_ to CO_2_ as the rate determining step in the mechanism. Thus, our impedance data support the fact that the step represented by eqn (3) is not determining the overall electrocatalytic process, but it is the methanol dehydrogenation step (eqn (2)) the key kinetic reaction in the mechanism.^50^

**Fig. 7:**
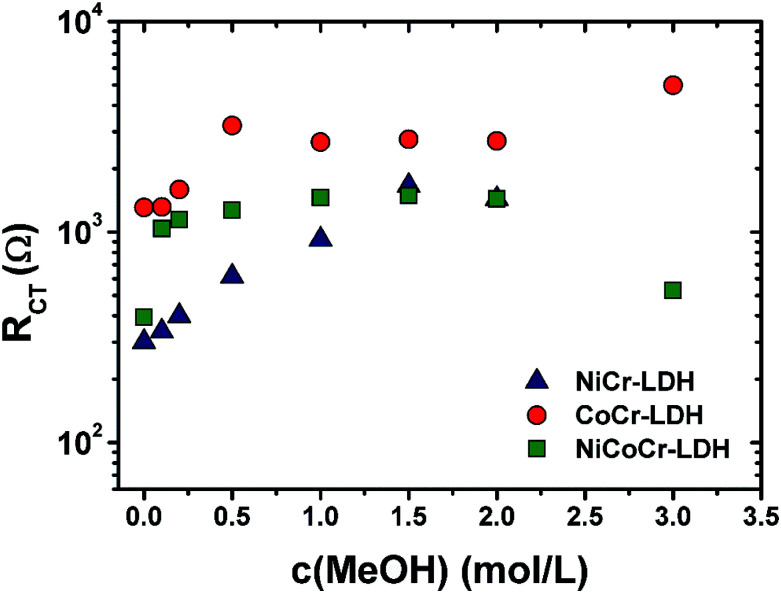
Variation of the charge transfer resistance (*R*_CT_) with different methanol concentrations for NiCr-LDH, CoCr-LDH and NiCoCr-LDH.

The catalytic activity of these catalysts is embodied in the *R*_CT_ parameter, and shall be given special attention in this discussion. The evolution of *R*_CT_*vs.* methanol concentration is depicted in Fig. 7; all other EIS fit parameters are presented in Table S1.† Consistently, the CoCr-LDH material exhibited the highest value of *R*_CT_, and therefore the poorest charge transfer kinetics. Regarding the other two nanohybrids LDH, for methanol concentrations lower than 1 M, the lowest values of *R*_CT_ and therefore the fastest heterogeneous reaction were observed for the NiCr-LDH solid. At the highest concentrations (1.5 and 2.0 M), the catalytic activity of NiCr-LDH and NiCoCr-LDH were similar as expressed by the similar values of *R*_CT_.

Particularly, in the case of the NiCr-LDH nanohybrid, at the highest concentration of methanol there was a sudden change of the shape of EIS, since the semicircle developed in the second quadrant and thus the *R*_ads_ value was negative (see Fig. S4†). This dramatic change in the spectrum is related to a change in the operating mechanism, whose rate determining step shifts from methanol dehydrogenation step (eqn (2), observed for all other conditions) to the oxidation of (CO)_ads_ (eqn (3)), which operates only at the surface of NiCr-LDH material at very high methanol concentrations.

The overall reactivity order, where the more reactive species is the one exhibiting the lowest value of *R*_CT_, was the following: NiCr-LDH > NiCoCr-LDH > CoCr-LDH as depicted in Fig. 7 for the oxidation reaction at 0.6 V using 2 M methanol as a representative example. This might be due not only to the intrinsic electrocatalytic activity of the material, but also to the highest pore width (*cf.*Table 1) which would enable an easier access of reactive species to the catalytic sites at the electrode|electrolyte interface.^8^

## 4. Conclusion

This study aims to find non-expensive catalyst with good performance and stability for methanol oxidation fuel cells. Using the co-precipitation method, three nanohybrids CoCr-LDH, NiCoCr-LDH and NiCr-LDH were prepared. All the prepared nanohybrids presented mesoporous structures and the NiCr-LDH had the highest pore width. Optimum tests could be achieved to detect the most efficient nanohybrid. The obtained plots from the CV tests showed superior oxidation of methanol activities with better stability for the NiCr-LDH material. The obtained current density using NiCr-LDH was 7.02 mA cm^−2^ at 60 mV s^−1^ using 3 M methanol. Where it exhibits the more negative onset potential 0.35 V. This might be due to the reversible one-electron redox couple nature of nickel and the pore width value which play the main role for its electrocatalytic activity by enabling an easier access of reactive species to the active sites. Furthermore, the kinetic analysis by EIS revealed that the NiCr-LDH material exhibited the lowest values of charge transfer resistance, which is consistent to the lowest observed over-potential for methanol oxidation.

## Conflicts of interest

There are no conflicts to declare.

## Supplementary Material

RA-009-C9RA01270B-s001

## References

[cit1] Habibi B., Ghaderi S. (2017). Electrosynthesized Ni-Al Layered Double Hydroxide-Pt Nanoparticles as an Inorganic Nanocomposite and Potentate Anodic Material for Methanol Electrooxidation in Alkaline Media. Bull. Chem. React. Eng. Catal..

[cit2] Luo S., Qian L., Liao M., Hu X., Xiao D. (2017). Surface and interface engineering of CoNi layered double hydroxides for efficient methanol oxidation reaction. RSC Adv..

[cit3] Zhao S., Yan L., Luo H., Mustain W., Xu H. (2018). Recent Progress and Perspectives of Bifunctional Oxygen Reduction/Evolution Catalyst Development for Unitized Regenerative Anion Exchange Membrane Fuel Cells. Nano Energy.

[cit4] Huang W., Wang H., Zhou J., Wang J., Duchesne P. N., Muir D., Zhang P., Han N., Zhao F., Zeng M., Zhong J., Jin C., Li Y., Lee S. T., Dai H. (2015). Highly active and durable methanol oxidation electrocatalyst based on the synergy of platinum-nickel hydroxide-graphene. Nat. Commun..

[cit5] Khouchaf A., Takky D., Chbihi M. E. M., Benmokhtar S. (2016). Electrocatalytic oxidation of methanol on glassy carbon electrode modified by metal ions (copper and nickel) dispersed into polyaniline film. J. Mater. Sci. Chem. Eng..

[cit6] Liu H., Song C., Zhang L., Zhang J., Wang H., Wilkinson D. P. (2006). A review of anode catalysis in the direct methanol fuel cell. J. Power Sources.

[cit7] Abdullah N., Kamarudin S. K., Shyuan L. K. (2018). Novel Anodic Catalyst Support for Direct Methanol Fuel Cell: Characterizations and Single-Cell Performances. Nanoscale Res. Lett..

[cit8] Qian L., Gu L., Yang L., Yuan H., Xiao D. (2013). Direct growth of NiCo 2 O 4 nanostructures on conductive substrates with enhanced electrocatalytic activity and stability for methanol oxidation. Nanoscale.

[cit9] El-Deeb M. M., El Rouby W. M. A., Abdelwahab A., Farghali A. A. (2018). Effect of pore geometry on the electrocatalytic performance of nickel cobaltite/carbon xerogel nanocomposite for methanol oxidation. Electrochim. Acta.

[cit10] Cao J., Chen H., Zhang X., Zhang Y., Liu X. (2018). Graphene-supported platinum/nickel phosphide electrocatalyst with improved activity and stability for methanol oxidation. RSC Adv..

[cit11] Zhang F., Wang Z., Xu K. Q., Xia J., Liu Q., Wang Z. (2018). Highly dispersed ultrafine Pt nanoparticles on nickel-cobalt layered double hydroxide nanoarray for enhanced electrocatalytic methanol oxidation. Int. J. Hydrogen Energy.

[cit12] Zhang G., Yang Z., Zhang W., Wang Y. (2017). Nanosized Mo-doped CeO2 enhances the electrocatalytic properties of the Pt anode catalyst in direct methanol fuel cells. J. Mater. Chem. A..

[cit13] Huang W., Wang H., Zhou J., Wang J., Duchesne P. N., Muir D., Zhang P., Han N., Zhao F., Zeng M. (2015). Highly active and durable methanol oxidation electrocatalyst based on the synergy of platinum–nickel hydroxide–graphene. Nat. Commun..

[cit14] Das S., Dutta K., Kundu P. P., Bhattacharya S. K. (2018). Nanostructured Polyaniline: An Efficient Support Matrix for Platinum-Ruthenium Anode Catalyst in Direct Methanol Fuel Cell. Fuel Cells.

[cit15] Abdel-Karim R., Ramadan M., El-Raghy S. M. (2018). Morphology and Electrochemical Characterization of Electrodeposited Nanocrystalline Ni-Co Electrodes for Methanol Fuel Cells. J. Nanomater..

[cit16] Lonkar S. P., Raquez J.-M., Dubois P. (2015). One-pot microwave-assisted synthesis of graphene/layered double hydroxide (LDH) nanohybrids. Nano-Micro Lett..

[cit17] Anantharaj S., Karthick K., Kundu S. (2017). Evolution of layered double hydroxides (LDH) as high performance water oxidation electrocatalysts: A review with insights on structure, activity and mechanism. Materials Today Energy.

[cit18] Wang Q., O'Hare D. (2012). Recent advances in the synthesis and application of layered double hydroxide (LDH) nanosheets. Chem. Rev..

[cit19] Elgiddawy N., Essam T. M., El Rouby W. M. A., Raslan M., Farghali A. A. (2017). New approach for enhancing Chlorella vulgaris biomass recovery using ZnAl-layered double hydroxide nanosheets. J. Appl. Phycol..

[cit20] Chowdhury P. R., Bhattacharyya K. G. (2017). Ni/Co/Ti layered double hydroxide for highly efficient photocatalytic degradation of Rhodamine B and Acid Red G: a comparative study. Photochem. Photobiol. Sci..

[cit21] El-Shahawy A. A. G., El-Ela F. I. A., Mohamed N. A., Eldine Z. E., El Rouby W. M. A. (2018). Synthesis and evaluation of layered double hydroxide/doxycycline and cobalt ferrite/chitosan nanohybrid efficacy on gram positive and gram negative bacteria. Mater. Sci. Eng., C.

[cit22] Fahim H. A., Rouby W. M. A. E., El-Gendy A. O., Khairalla A. S., Naguib I. A., Farghali A. A. (2017). Enhancement of the productivity of the potent bacteriocin avicin A and improvement of its stability using nanotechnology approaches. Sci. Rep..

[cit23] Sayed R. A., El Hafiz S. E. A., Gamal N., GadelHak Y., El Rouby W. M. A. (2017). Co-Fe layered double hydroxide decorated titanate nanowires for overall photoelectrochemical water splitting. J. Alloys Compd..

[cit24] Valente J. S., Figueras F., Gravelle M., Kumbhar P., Lopez J., Besse J.-P. (2000). Basic properties of the mixed oxides obtained by thermal decomposition of hydrotalcites containing different metallic compositions. J. Catal..

[cit25] Sarfraz M., Shakir I. (2017). Recent advances in layered double hydroxides as electrode materials for high-performance electrochemical energy storage devices. Journal of Energy Storage.

[cit26] Barakat N. A. M., Motlak M., Nassar M. M., Abdelkareem M. A., Mahmoud M. S., El-Newehy M. H., Moustafa H. M. (2015). From secondary to primary role in alkaline fuel cells: co-decorated graphene as effective catalyst for ethanol oxidation. ECS Electrochem. Lett..

[cit27] Ye W., Fang X., Chen X., Yan D. (2018). A three-dimensional nickel–chromium layered double hydroxide micro/nanosheet array as an efficient and stable bifunctional electrocatalyst for overall water splitting. Nanoscale.

[cit28] Dong C., Yuan X., Wang X., Liu X., Dong W., Wang R., Duan Y., Huang F. (2016). Rational design of cobalt-chromium layered double hydroxide as a highly efficient electrocatalyst for water oxidation. J. Mater. Chem. A..

[cit29] Zhu H., Liu Q., Li Z., Liu J., Jing X., Zhang H., Wang J. (2015). Synthesis of exfoliated titanium dioxide nanosheets/nickel–aluminum layered double hydroxide as a novel electrode for supercapacitors. RSC Adv..

[cit30] Ganley J. C., Karikari N. K., Raghavan D. (2010). Performance enhancement of alkaline direct methanol fuel cells by Ni/Al layered double hydroxides. J. Fuel Cell Sci. Technol..

[cit31] Vlamidis Y., Fiorilli S., Giorgetti M., Gualandi I., Scavetta E., Tonelli D. (2016). Role of Fe in the oxidation of methanol electrocatalyzed by Ni based layered double hydroxides: X-ray spectroscopic and electrochemical studies. RSC Adv..

[cit32] Ruan X., Chen Y., Chen H., Qian G., Frost R. L. (2016). Sorption behavior of methyl orange from aqueous solution on organic matter and reduced graphene oxides modified Ni-Cr layered double hydroxides. Chem. Eng. J..

[cit33] El Rouby W. M. A., El-Dek S. I., Goher M. E., Noaemy S. G. (2018). Efficient water decontamination using layered double hydroxide beads nanocomposites. Environ. Sci. Pollut. Res..

[cit34] Djebbi M. A., Braiek M., Namour P., Amara A. B. H., Jaffrezic-Renault N. (2016). Layered double hydroxide materials coated carbon electrode: New challenge to future electrochemical power devices. Appl. Surf. Sci..

[cit35] Tan X., Lan H., Xie H., Zhou G., Jiang Y. (2017). Role of surface oxygen species of mesoporous CeCu oxide catalyst in OVOCs catalytic combustion. J. Environ. Chem. Eng..

[cit36] Leofanti G., Padovan M., Tozzola G., Venturelli B. (1998). Surface area and pore texture of catalysts. Catal. Today.

[cit37] Intarasiri S., Ratana T., Sornchamni T., Phongaksorn M., Tungkamani S. (2017). Effect of pore size diameter of cobalt supported catalyst on gasoline-diesel selectivity. Energy Procedia.

[cit38] Song L., Sun Z., Duan L., Jiang S., Rees L. V. C. (2004). Investigation of adsorption hysteresis in microporous materials. Stud. Surf. Sci. Catal..

[cit39] Zeng Y., Prasetyo L., Tan S. J., Fan C., Do D. D., Nicholson D. (2017). On the hysteresis of adsorption and desorption of simple gases in open end and closed end pores. Chem. Eng. Sci..

[cit40] Ramírez A., Sierra L., Mesa M., Restrepo J. (2005). Simulation of nitrogen adsorption–desorption isotherms. Hysteresis as an effect of pore connectivity. Chem. Eng. Sci..

[cit41] Zhang L., Hui K. N., San Hui K., Lee H. (2015). Facile synthesis of porous CoAl-layered double hydroxide/graphene composite with enhanced capacitive performance for supercapacitors. Electrochim. Acta.

[cit42] Sheikh A. M., Abd-Alftah K. E.-A., Malfatti C. F. (2014). On reviewing the catalyst materials for direct alcohol fuel cells (DAFCs). Energy.

[cit43] Xu J., Gai S., He F., Niu N., Gao P., Chen Y., Yang P. (2014). Reduced graphene oxide/Ni 1 − x Co x Al-layered double hydroxide composites: preparation and high supercapacitor performance. Dalton Trans..

[cit44] Danaee I., Jafarian M., Forouzandeh F., Gobal F., Mahjani M. G. (2009). Electrochemical impedance studies of methanol oxidation on GC/Ni and GC/NiCu electrode. Int. J. Hydrogen Energy.

[cit45] Mohamed I., Motlak M., Obaid M., Alsoufi M. S., Bawazeer T. M., Mohamed A. F., Barakat N. A. M. (2017). Co/Cr-Decorated Carbon Nanofibers as Novel and Efficacious Electrocatalyst for Ethanol Oxidation in
Alkaline Medium. J. Nanosci. Nanotechnol..

[cit46] Zhang G., Li Y., Zhou Y., Yang F. (2016). NiFe Layered-Double-Hydroxide-Derived NiO-NiFe2O4/Reduced Graphene Oxide Architectures for Enhanced Electrocatalysis of Alkaline Water Splitting. ChemElectroChem.

[cit47] Kabbabi A., Faure R., Durand R., Beden B., Hahn F., Leger J.-M., Lamy C. (1998). In situ FTIRS study of the electrocatalytic oxidation of carbon monoxide and methanol at platinum–ruthenium bulk alloy electrodes. J. Electroanal. Chem..

[cit48] Guo J., Mao Z., Xu J. (2003). Studies on the electrochemical behavior of polymer electrolyte membrane fuel cell (PEMFC) by AC impedance method. Chem. J. Chin. Univ..

[cit49] Hsing I.-M., Wang X., Leng Y.-J. (2002). Electrochemical Impedance Studies of Methanol Electro-oxidation on Pt/C Thin Film Electrode. J. Electrochem. Soc..

[cit50] Shahrokhian S., Rezaee S. (2018). Vertically standing Cu2O nanosheets promoted flower-like PtPd nanostructures supported on reduced graphene oxide for methanol electro-oxidation. Electrochim. Acta.

[cit51] Ghosh S., Raj C. R. (2010). Facile in situ synthesis of multiwall carbon nanotube supported flowerlike pt nanostructures: An efficient electrocatalyst for fuel cell application. J. Phys. Chem. C.

[cit52] Seland F., Tunold R., Harrington D. A. (2006). Impedance study of methanol oxidation on platinum electrodes. Electrochim. Acta.

[cit53] Kulikovsky A. A., Löhmer A., Wippermann K. (2013). The features of a direct methanol fuel cell cathode impedance due to methanol crossover: Modeling and experiment. Electrochim. Acta.

[cit54] Teliz E., Díaz V., Zinola C. F. (2014). The enhancement of methanol oxidation electrocatalysis at low and high overpotentials. Electrochim. Acta.

[cit55] Li Z., Yang R., Li B., Yu M., Li D., Wang H., Li Q. (2017). Controllable synthesis of graphene/NiCo2O4 three-dimensional mesoporous electrocatalysts for efficient methanol oxidation reaction. Electrochim. Acta.

[cit56] Silva C. D., Morais L. H., Gonçalves R., Matos R., Souza G. L. C., Freitas R. G., Pereira E. C. (2018). The methanol and CO electro-oxidation onto Pt pc/Co/Pt metallic multilayer nanostructured electrodes: An experimental and theoretical approach. Electrochim. Acta.

[cit57] Holm T., Dahlstrøm P. K., Sunde S., Seland F., Harrington D. A. (2019). Dynamic electrochemical impedance study of methanol oxidation at Pt at elevated temperatures. Electrochim. Acta.

[cit58] Lyons M. E. G., Brandon M. P. (2009). The significance of electrochemical impedance spectra recorded during active oxygen evolution for oxide covered Ni, Co and Fe electrodes in alkaline solution. J. Electroanal. Chem..

[cit59] Schulz T., Weinmüller C., Nabavi M., Poulikakos D. (2010). Electrochemical impedance spectroscopy analysis of a thin polymer film-based micro-direct methanol fuel cell. J. Power Sources.

[cit60] Müller J. T., Urban P. M., Hölderich W. F. (1999). Impedance studies on direct methanol fuel cell anodes. J. Power Sources.

[cit61] Chung D. Y., Lee K. J., Sung Y. E. (2016). Methanol electro-oxidation on the Pt surface: Revisiting the cyclic voltammetry interpretation. J. Phys. Chem. C.

