# Supplementary material for: Nanohybrid layered double hydroxide materials as efficient catalysts for methanol electrooxidation
Source: RSC Adv. 2019 May 1;9(24):13503–14. doi: 10.1039/c9ra01270b (PMC9063938; doi:10.1039/c9ra01270b)
Supplement: RA-009-C9RA01270B-s001 [file RA-009-C9RA01270B-s001.pdf]

Electronic Supplementary Material (ESI) for RSC Advances.
This journal is © The Royal Society of Chemistry 2019

Supporting materials

Nanohybrid layered double hydroxides materials as efficient catalysts for methanol electrooxidation

Shimaa Gamil[1], Waleed M. A. El Rouby[2]*, Manuel Antuch[3], I.T. Zedan[1]

[1] Renewable Energy Science and Engineering Department, Faculty of Postgraduate Studies for Advanced Science, Beni-Suef University, 62511 Beni-Suef, Egypt.

[2] Materials Science and Nanotechnology Department, Faculty of Postgraduate Studies for Advanced Science, Beni-Suef University, 62511 Beni-Suef, Egypt.

[3] Paris-Sud University, ICMMO-Eriée, UMR CNRS 8182, 91405 Orsay, France.

* Corresponding author e-mail: waleedmohamedali@psas.bsu.edu.eg

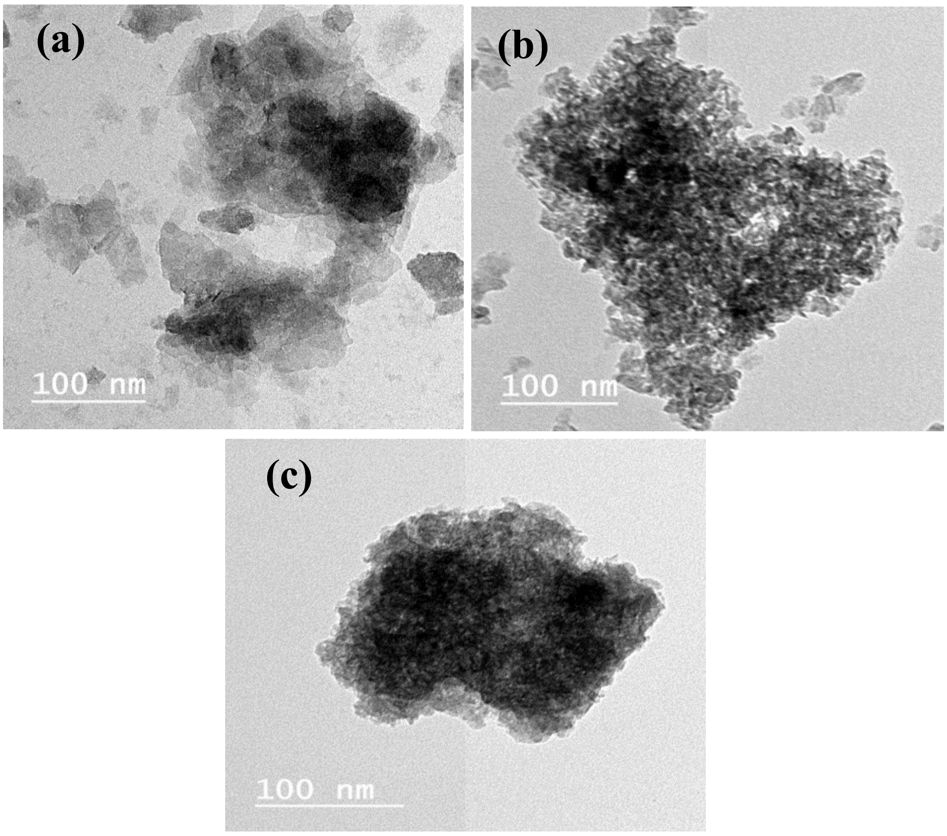

Fig. S1; TEM images of hybrid (a) NiCr- LDH, (b) CoCr- LDH and (c) NiCoCr-LDH

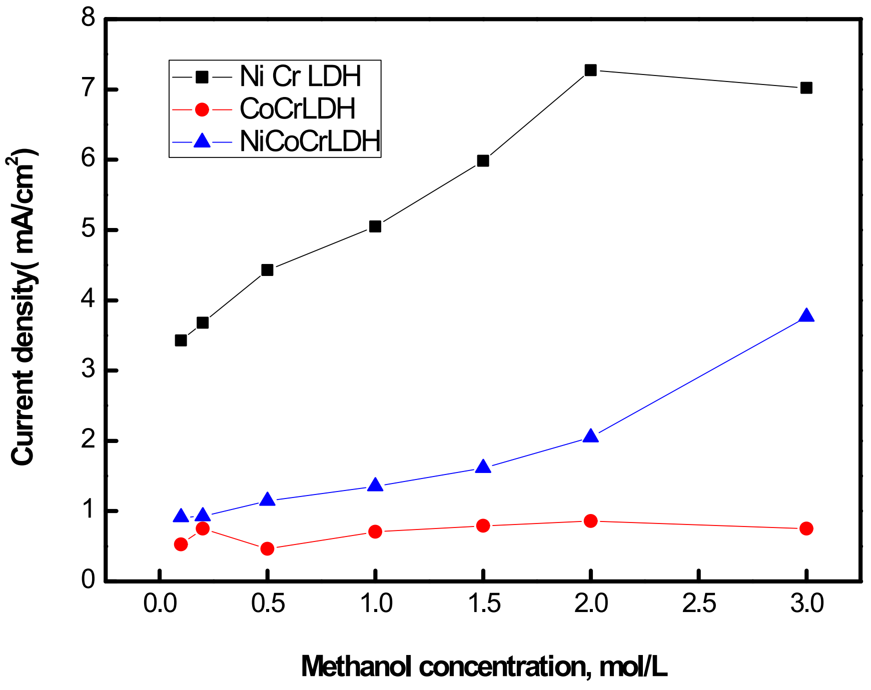

Fig. S2; Variation of current density with different methanol concentrations for Ni Cr-LDH, Co Cr-LDH and Ni Co Cr-LDH

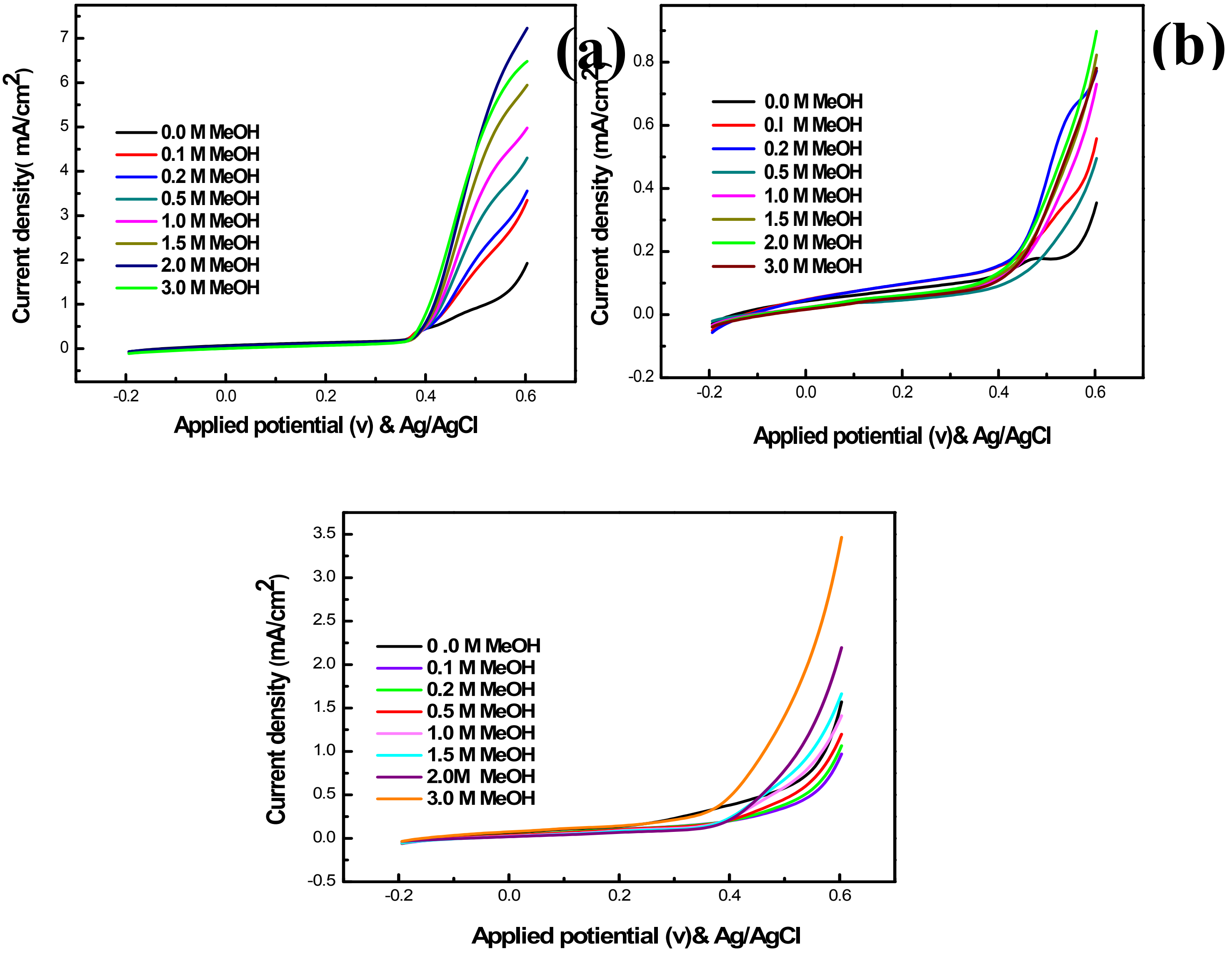

Fig.S3; The onset potentials for the hybrids (a) NiCr- LDH and (b) CoCr- LDH (c) NiCoCr- LDH with different methanol concentrations, at 60 Mvs^{-1} scan rate.

Table S1. Fitting results of the Impedance Spectra. Resistances are expressed in

NiCr-LDH						
c(MeOH)	R_{CT}	R_{ads}	Q_{ads}	Q_{dl}	n_1	n_2
0	3.00E+02	1.02E+02	3.99E-04	2.50E-05	0.85	0.85
0.1	3.38E+02	1.31E+02	2.78E-04	2.27E-05	0.85	0.85
0.2	3.99E+02	1.29E+02	2.17E-04	2.22E-05	0.85	0.85
0.5	6.15E+02	1.18E+02	9.00E-04	2.41E-05	0.85	0.85
1	9.20E+02	2.76E+02	7.02E-04	2.40E-05	0.85	0.85
1.5	1.66E+03	1.44E+03	4.75E-04	2.74E-05	0.85	0.85
2	1.44E+03	1.46E+03	6.63E-04	2.88E-05	0.85	0.85
3	1.42E+02	-1.7E+03	1.03E-03	2.94E-05	0.80	0.95

CoCr-LDH						
c(MeOH)	R_{CT}	R_{ads}	Q_{ads}	Q_{dl}	n_1	n_2
0	1.31E+03	3.14E+02	2.00E-04	1.37E-05	0.86	0.85
0.1	1.32E+03	2.63E+02	3.43E-04	1.43E-05	0.86	0.85
0.2	1.59E+03	4.29E+02	4.93E-04	1.53E-05	0.85	0.85
0.5	3.20E+03	6.31E+02	1.67E-04	2.64E-05	0.80	0.80
1	2.67E+03	5.46E+02	2.52E-04	2.71E-05	0.80	0.80
1.5	2.76E+03	4.17E+02	4.99E-04	2.71E-05	0.80	0.80
2	2.71E+03	5.00E+02	3.17E-04	2.57E-05	0.80	0.80
3	4.98E+03	5.44E+02	2.45E-03	2.46E-05	0.80	0.80

NiCoCr-LDH						
c(MeOH)	R_{CT}	R_{ads}	Q_{ads}	Q_{dl}	n_1	n_2
0	3.94E+02	1.76E+01	1.00E-04	8.72E-05	0.86	0.98
0.1	1.04E+03	5.70E+01	1.62E-03	3.23E-05	0.80	0.82
0.2	1.14E+03	3.72E+01	1.35E-03	2.87E-05	0.80	0.81
0.5	1.27E+03	3.89E+01	1.01E-04	2.36E-05	0.81	1.00
1	1.45E+03	2.35E+01	1.99E-04	1.98E-05	0.83	1.00
1.5	1.49E+03	5.09E+01	3.00E-03	1.78E-05	0.86	0.80
2	1.44E+03	5.60E+01	1.76E-03	1.79E-05	0.88	0.80
3	5.27E+02	9.68E+01	1.00E-04	5.56E-05	0.86	1.00

Ohms, while Q has units of $F/s^{(1-n)}cm^2$ where n is the CPE exponent.

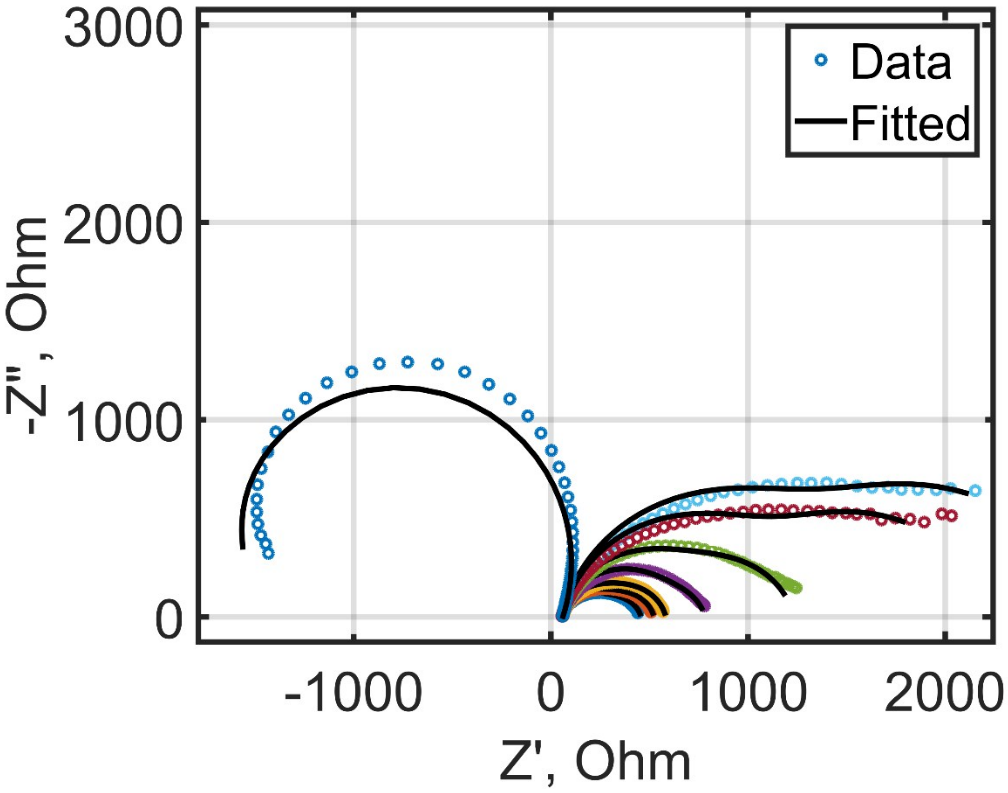

Fig. S4; EIS LDH NiCr the for fits and data material

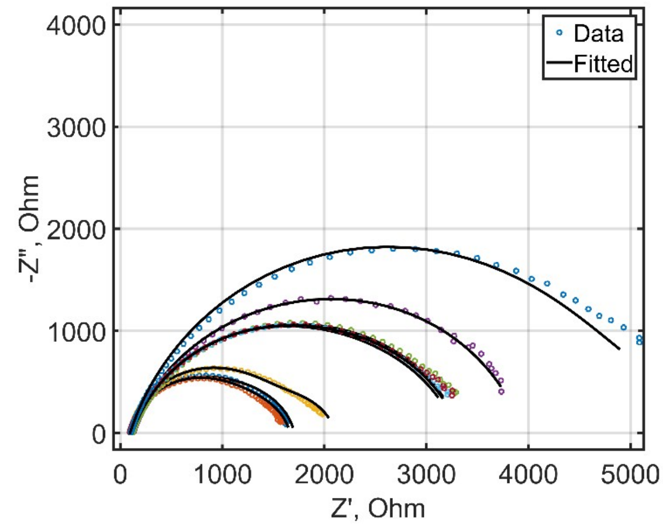

Fig. S5; LDH CoCr the for fit and data EIS material

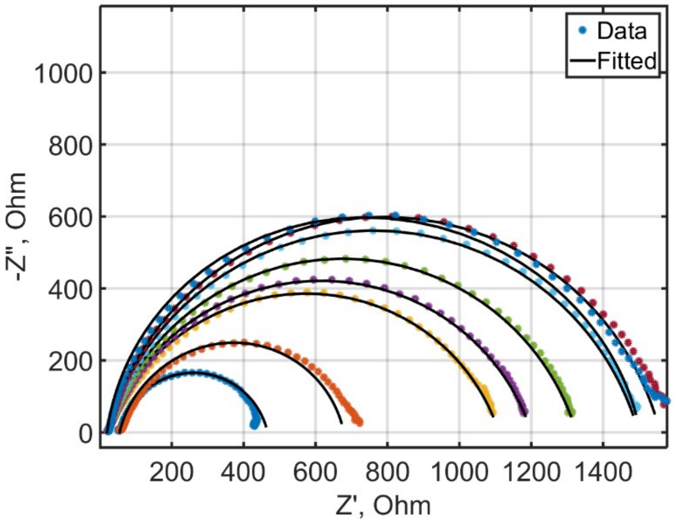

Fig. S6;material LDH NiCoCr the for fits and data EIS
